# Rapid and efficient high-performance liquid chromatography analysis of *N*-nitrosodimethylamine impurity in valsartan drug substance and its products

**DOI:** 10.1038/s41598-019-48344-5

**Published:** 2019-08-14

**Authors:** Sayaka Masada, Genichiro Tsuji, Ryoko Arai, Nahoko Uchiyama, Yosuke Demizu, Tomoaki Tsutsumi, Yasuhiro Abe, Hiroshi Akiyama, Takashi Hakamatsuka, Ken-ichi Izutsu, Yukihiro Goda, Haruhiro Okuda

**Affiliations:** National Institute of Health Sciences, 3-25-26 Tonomachi, Kawasaki-ku, Kawasaki, Kanagawa 210-9501 Japan

**Keywords:** Small molecules, Chemical tools, Chemical safety

## Abstract

In July 2018, certain valsartan-containing drugs were voluntary recalled in Japan owing to contamination with *N*-nitrosodimethylamine (NDMA), a probable human carcinogen. In this study, an HPLC method was developed for the quantitative detection of NDMA simultaneously eluted with valsartan. Good linearity with a correlation coefficient (R^2^) > 0.999 was achieved over the concentration range of 0.011–7.4 µg/mL. The limits of detection and quantification were 0.0085 μg/mL and 0.0285 μg/mL, respectively. When the recalled valsartan samples were subjected to this method, the observed NDMA contents were in agreement with the reported values, indicating that our method achieved sufficient linearity, accuracy, and precision to detect NDMA in valsartan drug substances and products. Moreover, six samples (valsartan drug substances and tablet formulations), which had a possibility for NDMA contamination, were analyzed; none of the samples contained NDMA at detectable levels. Our method would be useful for the rapid screening and quantification of NDMA impurity in valsartan drug substances and products.

## Introduction

Valsartan-containing drugs contain the active pharmaceutical ingredient (API) valsartan. Valsartan [(2*S*)-3-Methyl-2-(*N*-{[2′-(1*H*-tetrazol-5-yl)biphenyl-4-yl]methyl}pentanamido)butanoic acid], an angiotensin II receptor antagonist, is mainly used for the treatment of hypertension and congestive heart failure. On 6^th^ July 2018, the Ministry of Health, Labour and Welfare (MHLW) in Japan released that *N*-nitrosodimethylamine (NDMA) was detected as an impurity in valsartan-containing drugs whose API was supplied by Zhejiang Huahai Pharmaceutical in China. Simultaneously, ASKA Pharmaceutical Co., Ltd. in Japan announced a voluntary product recall of valsartan-containing drugs, because they found that there could be a risk of contamination of NDMA in the API purchased from Zhejiang Huahai Pharmaceutical^1^. In addition, the MHLW recently notified that NDMA impurity in valsartan drug substances should not exceed 0.599 ppm^2^.

**Figure 1:**
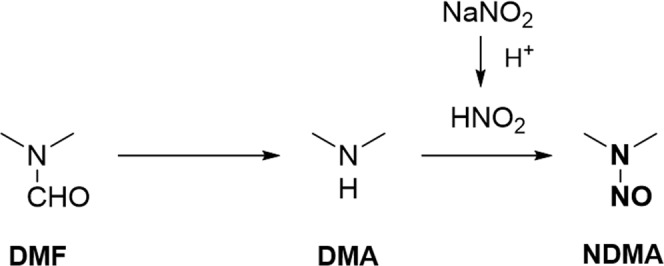
Prospective mechanism of NDMA production during the manufacturing process of valsartan (DMF: *N*,*N*-Dimethylformamide; DMA: Dimethylamine; NDMA: *N*-Nitrosodimethylamine).

NDMA is classified as a probable human carcinogen based on results from laboratory animal tests^3–5^ and is listed under WHO/IARC group 2A and EPA group B2^6–8^. NDMA contamination was thought to be caused by the following changes in the production process of valsartan API^9,10^: NDMA was generated during the tetrazole-formation step owing to the presence of dimethylamine as an impurity or a degradant in *N*,*N*-dimethylformamide (DMF) solvent and the presence of nitrous acid generated from sodium nitrite under acidic conditions (Fig. 1).

As of September 2018, valsartan-containing products whose APIs were supplied from Zhejiang Huahai Pharmaceutical Co., Ltd., Zhejiang Tianyu Pharmaceutical Co., Ltd., Hetero Labs Ltd., and Zhuhai Rundu Pharmaceutical Co., Ltd. have been recalled in more than 20 countries, including the EU and USA^11–16^. Regulatory bodies, European Medicines Agency (EMA), U.S. Food and Drug Administration (FDA), and the MHLW, cooperatively worked to handle the situation and take necessary measures to mitigate patient risk while estimating the risk of cancer due to NDMA-contaminated valsartan products^17,18^. Government laboratories, including National Institute of Health Sciences in Japan, parallelly prepared methods to analyze NDMA in valsartan APIs and products in each market.

The API valsartan is listed in the Japanese Pharmacopoeia (JP)^19^ with methods for testing identity, purity, and assay; it is also listed in the US and European Pharmacopoeias^20,21^. A purity test mainly focusses on expected impurities from synthesis and/or degradation. The purity test for valsartan focusses on heavy metals and related substances; however, these pharmacopoeias have never mentioned the need for testing NDMA as an impurity.

NDMA is mainly generated in foods and drinks after processing at an elevated temperature^22–24^. It is also detected as a disinfection by-product in ground and drinking water^25–27^. As the toxicity of NDMA is manifested even at μg/kg levels^28,29^, sensitive and specific methods were developed for the determination of NDMA at trace level. Gas chromatography–mass spectrometry (GC-MS) is the most frequently employed technique for NDMA analyses^30–32^. In addition, several methods using liquid chromatography–mass spectrometry (LC-MS) or LC-MS/MS have been reported in scientific literature^33–35^. However, only few studies have reported NDMA analysis using conventional high-performance liquid chromatography (HPLC)^36^, especially in drugs. HPLC is the most popular technique for quality control of APIs and products in routine analysis, and it is preferable if NDMA impurity is simultaneously detected with drug substances by a single HPLC analysis. Thus, it is important to develop a fast and simple analytical method for NDMA in drugs by using HPLC.

In view of these situations, we tried to develop an HPLC method for the simultaneous detection of NDMA and valsartan. We analyzed valsartan drug substances and its products, including recalled samples, and confirmed the accuracy and precision of the method. This study provides a simple and accurate method for the quantification of NDMA impurity in valsartan products.

## Results and Discussion

### HPLC method development

**Figure 2:**
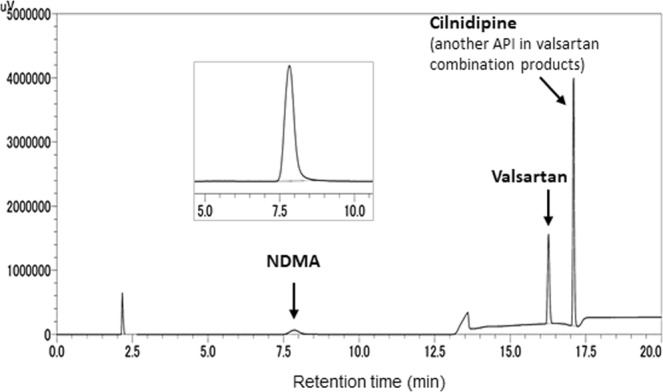
HPLC chromatograms of a mixture of reference standards.

To establish a practical method for the simultaneous detection of NDMA and valsartan, we first assayed each standard solution according to the HPLC condition for the quantitative assay for valsartan API and its tablet formulation defined in the JP^19^. When each standard solution of valsartan and NDMA was assayed following the modified JP method using isocratic mobile conditions, it was difficult to identify the peak of NDMA as it eluted during the void time (2.5 min) even with a flow rate of 0.80 mL/min, whereas the peak of valsartan was clearly detected at around 11 min (data not shown). Then, we developed a gradient elution program using a water-acetonitrile mobile phase containing 0.1% formic acid to detect NDMA and valsartan simultaneously within 30 min. Under this condition, peaks of NDMA and valsartan were successfully detected at 7.8 and 16.3 min, respectively (Fig. 2). Moreover, we confirmed the simultaneous detection of cilnidipine, another API in valsartan combination products, at 17.1 min under the same condition.

To assess the linearity of the developed method, we prepared a calibration plot using 10 concentration points of NDMA in the range of 0.0111–7.4 μg/mL (0.15–100 μM) and constructed the calibration curve for quantification. The correlation coefficient (R^2^) of the calibration curve was over 0.999. The limits of detection (LODs) and quantification (LOQs) were 0.0085 μg/mL (at a S/N ratio of 3) and 0.0285 μg/mL (at a S/N ratio of 10), respectively. The standardized limits of NDMA impurity in valsartan drug substances, which was set as 0.559 ppm by the MHLW^2^, was equivalent to 0.02995 μg/mL when 0.1 g of sample was extracted with 2 mL methanol. This concentration was almost equal to the LOQ. Recently, we reported a GC-MS method for the detection of NDMA in valsartan drug substance and products with much lower LOD (0.001 μg/mL of NDMA corresponded to a S/N of 3)^37^. However, this method needed the isotopic internal standard (NDMA-*d*_6_) and multiple extraction steps as required for other MS-based methods. Generally, an HPLC method is low-cost and more suitable to routine analyses. Thus, the developed method would be useful for the rapid screening of NDMA contamination in valsartan drug substances with sufficient sensitivity.

### Quantification of NDMA impurity in the recalled valsartan samples

**Figure 3:**
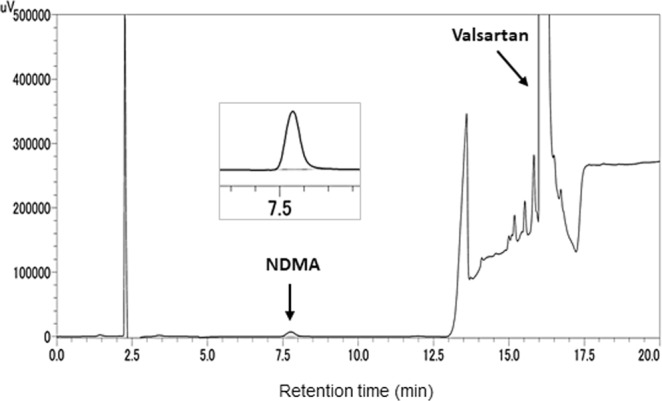
HPLC chromatograms of a sample solution of valsartan drug substances produced by Zhejiang Huahai Pharmaceutical Co., Ltd.

Following the confirmation of linearity, we evaluated NDMA contents in the recalled valsartan samples using the developed HPLC method. VALSARTAN TABLETS 80 mg [AA] and its substances were provided by ASKA Pharmaceutical Co., Ltd. through the MHLW. NDMA contents were estimated to be 36–74 ppm in the drug substances and 3.1–5.3 μg per tablet, respectively, based on GC-MS analysis according to the press release^13^. When a sample solution of drug substance (50 mg/mL in methanol) was analyzed using the developed method, the peak at 7.8 min was identified by comparing its retention time and UV spectrum with those of the NDMA reference standard (Fig. 3). NDMA content was calculated to be 54.7 μg/g, which was in agreement with the published value^1^. A sample solution of the commercial product (150 mg/mL in methanol) provided a similar chromatogram, and its NDMA content was determined to be 17.0 μg/g. The corresponding NDMA content was estimated to be 4.25 μg per tablet (250 mg), which was in agreement with the published data (Table 1)^1^.

**Table 1:** Comparison of NDMA contents observed by analysis of valsartan API and its tablets supplied by Zhejiang Huahai Pharmaceutical with previously reported values.

Product name	Manufacturer	Sample	Concentration of NDMA (μg/g)	NDMA contents (μg/tab)	Avg. ± SD	Reported NDMA value*
VALSARTAN TABLET 80 mg [AA]	ASKA Pharmaceutical Co., Ltd.	Substance	51.4	—	54.7 ± 0.56	58 (μg/g)
			52.1	—		
			51.1	—		
		Tablets	15.7	3.91	4.25 ± 0.10	4.2 (μg/tab)
			16.4	4.10		
			15.9	3.99		

*Previously determined values by GC-MS^1^.

### Recovery test

Successively, a recovery test was carried out by analyzing spiked samples in the same way. Forty microliters of 10 mM NDMA (29.6 μg) was added to powdered VALSARTAN TABLETS [AA] (300 mg), and the spiked sample solution fortified at 98.67 μg/g was prepared. Five replicates of the spiked samples and triplicates of blanks (samples not spiked) were analyzed, and the recovery rate was determined to be 96.5% with 0.67% relative standard deviation (RSD). These results indicate that the developed method has sufficient linearity, accuracy, and precision for the quantification of NDMA impurity in contaminated valsartan final products as well as its drug substances.

### HPLC assay on APIs and commercial valsartan products

Finally, we investigated NDMA content in APIs and commercially available valsartan-containing products in the same way. Four valsartan APIs and two tablets, including an original drug ATEDIO® Combination Tab. (containing 80 mg valsartan and 10 mg cilnidipine), were supplied by Zhejiang Tianyu Pharmaceutical Co. Ltd. When their sample solutions (50 mg/mL and 150 mg/mL in methanol for API and tablets, respectively) were analyzed, no peak was detected for NDMA in the APIs of VALSARTAN TABLET 80 mg [SAWAI] and VALSARTAN TABLET 80 mg [OHARA]. Any peaks other than those for valsartan and cilnidipine were not detected in the API and tablets of ATEDIO® Combination Tab. Although a small peak was observed at around 8 min on the HPLC chromatogram of VALSARTAN TABLET 80 mg [SANOFI], it had a retention time different from that of NDMA and was not detected in the API. Thus, NDMA contents in all the samples were indicated to be below the LOD (<0.17 μg/g for substances and <0.06 μg/g for tablets) and below the acceptance limit for NDMA (0.599 ppm) (Table 2). The EMA reported that NDMA concentrations in valsartan API from Zhejiang Tianyu Pharmaceutical Co., Ltd. were considerably lower than those from Zhejiang Huahai Pharmaceutical^14^, and only one batch of valsartan-containing drugs distributed in Germany was recalled^13^. NDMA was not detectable in the tested samples probably because the manufacturing process of valsartan APIs for Japanese companies could be different from that for other foreign companies. Hence, we successfully developed a practical method for the rapid screening and quantification of NDMA impurity in valsartan-containing products.

**Table 2:** Concentration of NDMA impurity in valsartan API and commercial products supplied by Zhejiang Tianyu Pharmaceutical.

Product name	Manufacturer	Sample	Concentration of NDMA (μg/g)
VALSARTAN TABLETS 80 mg [SAWAI]	SAWAI Pharmaceutical Co., Ltd.	Substance	Not detected (<0.17)
VALSARTAN TABLET 80 mg [OHARA]	OHARA Pharmaceutical Co., Ltd.	Substance	Not detected (<0.17)
VALSARTAN TABLETS 80 mg [SANOFI]	Nihon Pharmaceutical Industry Co., Ltd.	Substance	Not detected (<0.17)
		Tablets	Not detected (<0.06)
ATEDIO® Combination Tab.	EA Pharma Co., Ltd.	Substance	Not detected (<0.17)
		Tablets	Not detected (<0.06)

## Conclusion

A rapid and efficient HPLC method was developed for the quantitative detection of NDMA simultaneously eluted with valsartan. The method was found to have sufficient linearity, accuracy, and precision, and can be applied for the rapid screening and quantification of NDMA impurity in valsartan APIs and commercial products. This HPLC method would be useful for the quality control of APIs and products in routine analysis.

## Methods

### Reagents and materials

The commercial reagents of valsartan, cilnidipine, and NDMA with high purity (>98.0%, >98.0% and >99%, respectively) were purchased from Tokyo Chemical Industry Co., Ltd (Tokyo, Japan). APIs and its tablet formulations were provided by each company through the MHLW (Table 3).

**Table 3:** Valsartan APIs and its products analyzed in this study.

Product name	Manufacturer	Tested sample	Supplier
VALSARTAN TABLET 80 mg [AA]	ASKA Pharmaceutical Co., Ltd.	Substance	Zhejiang Huahai Pharmaceutical Co., Ltd.
		Tablets	
VALSARTAN TABLETS 80 mg [SAWAI]	SAWAI Pharmaceutical Co., Ltd.	Substance	Zhejiang Tianyu Pharmaceutical Co., Ltd.
VALSARTAN TABLET 80 mg [OHARA]	OHARA Pharmaceutical Co., Ltd.	Substance	
VALSARTAN TABLETS 80 mg [SANOFI]	Nihon Pharmaceutical Industry Co., Ltd.	Substance	
		Tablets	
ATEDIO® Combination Tab.	EA Pharma Co., Ltd.	Substance	
		Tablets	

Other reagents were of analytical grade.

### Sample preparation

NDMA (14.8 μL, Mw: 74.08, density: 1.005) was transferred into a 2-mL volumetric flask, dissolved to volume with methanol (NDMA = 7.4 mg/mL, corresponding to 100 mM), and diluted 100-fold to obtain the stock solution at 1 mM. The standard solution of NDMA was prepared by 10-fold dilution of the stock solution for HPLC analysis (100 μM). Valsartan (8.7 mg, Mw: 435.53) and cilnidipine (9.9 mg, Mw: 492.53) reagents were accurately weighed, individually transferred into a 2-mL volumetric flask, and dissolved to volume with methanol to obtain the stock solutions at 10 mM. Each stock solution was diluted 100-fold to a final concentration of 100 μM as standard solutions and filtered through a 0.45-μm Ultrafree-MC centrifugal filter unit (Millipore, Billerica, MA) before HPLC analysis.

The drug substance (100 mg) or powdered tablet (300 mg) was dissolved with 2 mL methanol and centrifuged at 5,000 rpm for 5 min. The supernatant was filtered through a 0.45-μm Ultrafree-MC centrifugal filter unit. Triplicate test samples for each commercial product were prepared from every press-through sheet.

As NDMA is a carcinogenic substance, its handling was carried out in accordance with Safety Data Sheet, and the preparation of samples containing NDMA was performed in a fume hood.

### HPLC analysis

HPLC method development and analyses were performed on a Shimadzu UFLC system comprising a binary gradient pump (LC-20AD), an autosampler (SIL-20AC), a column oven (CTO-20A), and a photodiode array detector (SPD-M20A) (Shimadzu, Tokyo, Japan). The first trial was carried out according to a modification of the method cited in the monographs of valsartan and its tablet in JP^19^. Briefly, 10 μL NDMA standard solution was assayed by isocratic elution with a mixture of water, acetonitrile, and acetic acid (100) (500: 500: 1) on an HPLC system equipped with Unison UK-C18 column (250 × 4.6 mm, 3 μm, Imtakt, Kyoto, Japan) at a flow rate 0.8 mL/min and detected at 235 nm. Although the wavelength of maximum absorption of NDMA standard solution was 228 nm, we set the detection wavelength at 235 nm to achieve detection at lower noise and better baseline stability. In the finalized condition, HPLC analysis was carried out on Inertsil ODS-3 column (150 × 4.6 mm, 5 μm, GL Science, Tokyo, Japan) at 30 °C with a mobile phase comprising water containing 0.1% formic acid (A) and acetonitrile containing 0.1% formic acid (B) and detected at 235 nm. The gradient elution started at 0% B in 10 min and increased linearly to 100% in 5 min at a flow rate of 1.0 mL/min. A 10-μL aliquot of each sample was injected three times, and the reproducibility of the result was confirmed. Each peak obtained from the test samples was identified by comparing its retention time and UV spectrum with those of the reference standard for valsartan, cilnidipine, and NDMA. Peak areas were determined by the automatic integration method. The standard solution of NDMA was diluted for preparing calibration solutions at 0.15, 0.2, 0.3, 0.4, 0.5, 1, 5, 10, 50, 100 μM with methanol. The calibration solutions were analyzed to plot a calibration curve, and its slope, intercept, and coefficient of determination were calculated.

### Recovery test

Forty microliters of 10 mM NDMA standard solution (740 μg/mL in methanol) was spiked to 300 mg of powdered VALSARTAN TABLETS [AA] and allowed to stand for 10 min. The spiked sample was dissolved with 1960 μL methanol and centrifuged at 5,000 rpm for 5 min. The supernatant was filtered through a 0.45-μm Ultrafree-MC centrifugal filter unit. The spiked sample was fortified to 98.67 μg/g with NDMA. Five replicates of spiked samples and three blanks (samples not spiked) were prepared and analyzed to determine the percentage of recovery.

## Data Availability

The datasets generated during and/or analyzed during the current study are available from the corresponding authors on reasonable request.
